# Integrating Individual-Based Indices of Contaminant Effects

**DOI:** 10.1100/tsw.2001.367

**Published:** 2001-11-29

**Authors:** Christopher L. Rowe, William A. Hopkins, Justin D. Congdon

**Affiliations:** University of Maryland Center for Environmental Science, Chesapeake Biological Laboratory, PO Box 38, Solomons, MD 20688, USA

**Keywords:** behavior, bullfrog, coal combustion wastes, deformities, ecotoxicology, energetics, heavy metals, metabolic rate, morphology, swimming speed, trace elements

## Abstract

Habitat contamination can alter numerous biological processes in individual organisms. Examining multiple individual-level responses in an integrative fashion is necessary to understand how individual health or fitness reflects environmental contamination. Here we provide an example of such an integrated perspective based upon recent studies of an amphibian (the bullfrog, Rana catesbeiana) that experiences several, disparate changes when larval development occurs in a trace element–contaminated habitat. First, we present an overview of studies focused on specific responses of individuals collected from, or transplanted into, a habitat contaminated by coal combustion residues (CCR). These studies have reported morphological, behavioral, and physiological modifications to individuals chronically interacting with sediments in the CCR-contaminated site. Morphological abnormalities in the oral and tail regions in contaminant-exposed individuals influenced other properties such as grazing, growth, and swimming performance. Behavioral changes in swimming activities and responses to stimuli appear to influence predation risk in the contaminant-exposed population. Significant changes in bioenergetics in the contaminated habitat, evident as abnormally high energetic expenditures for survival (maintenance) costs, may ultimately influence production pathways (growth, energy storage) in individuals. We then present a conceptual model to examine how interactions among the affected systems (morphological, behavioral, physiological) may ultimately bring about more severe effects than would be predicted if the responses were considered in isolation. A complex interplay among simultaneously occurring biological changes emerges in which multiple, sublethal effects ultimately can translate into reductions in larval or juvenile survival, and thus reduced recruitment of juveniles into the population. In systems where individuals are exposed to low concentrations of contaminants for long periods of time, research focused on one or few sublethal responses could substantially underestimate overall effects on individuals. We suggest that investigators adopt a more integrated perspective on contaminant-induced biological changes so that studies of individual-based effects can be better integrated into analyses of mechanisms of population change.

## INTRODUCTION

Exposure to contaminants in natural systems can bring about changes in organisms at molecular, cellular, tissue, and whole-individual levels. Changes in individuals as a whole represent the integration of significant effects occurring at lower levels of organization, and when individual processes (such as growth, survival probability, reproductive fitness) are modified by contaminants, ramifications at the population level may emerge. Yet, rarely have multiple, disparate individual-based responses been quantified in a given species chronically interacting with contaminants in an environmentally realistic setting. Given the diversity of potential responses by individuals experiencing chronic exposure to contaminant mixtures in the environment, an integration of numerous endpoints can provide a conceptual basis for considering overall influences on individual health or fitness.

In this synthesis, we bring together information from several recent studies examining individual responses of larval bullfrogs (*Rana catesbeiana*) measured in a site contaminated by solid coal combustion residue (CCR) to examine the ways in which these responses may interact to affect individuals *in toto*. The goal of this paper is to emphasize the utility of measuring multiple effects in individuals exposed to contaminants so that possible explanations for population level changes may be found.

The quantity of coal consumed worldwide is expected to increase by 27% over the period from 1990 to 2010[1]. As a result, solid CCR (a waste product of coal combustion) will be an important and increasing source of contaminants to natural environments. In the U.S. alone, nearly 109 million tonne of CCR are currently produced annually[2]. A substantial amount of CCR is recycled or dry-land filled, but 28% of ash produced in the U.S. is disposed of by pumping slurried ash into settling basins for passive removal of particulate matter prior to discharge of the surface waters into local watersheds[3]. Open settling basins used for CCR disposal are occupied by a variety of plants and animals. Because CCR characteristically is enriched in trace elements such as As, Cd, Cr, Cu, Hg, and Se, organisms that inhabit or frequently visit CCR disposal sites may accumulate high levels of these and other trace elements and heavy metals[4,5,6,7,8,9,10,11,12,13,14,15].

Since the mid-1990s, studies have been underway examining physiological and ecological responses of vertebrates and invertebrates to long-term exposure to CCR at a site in South Carolina. The site consists of two open settling basins (15 and 6 ha) and a drainage swamp (2 ha) that receive a CCR slurry from a coal-fired steam and electricity generating station (the ‘D-area facility’) located on the U.S. Department of Energy’s Savannah River Site. The slurry sequentially passes through the settling basins prior to the surface waters entering the drainage swamp. Outflow from the drainage swamp enters Beaver Dam Creek, which empties into the Savannah River about 2 km downstream. Compared to nearby reference sites, sediments, water, and biota in the disposal system and Beaver Dam Creek have elevated concentrations of a wide variety of trace elements and heavy metals derived from the ash[4,5,6,11,12,13,14,15,16,17,18]. While the D-Area site has been characterized with respect to contaminants for quite some time, only recently has the site been studied with respect to sublethal responses of biota.

Much work on biological responses to CCR has been conducted on premetamorphic life stages (embryos and larvae) of the bullfrog (*R. catesbeiana*). Bullfrogs are extremely useful as a bioindicator of responses to environmental contaminants. We chose to study bullfrogs in D-Area because: (1) large numbers of bullfrogs use the settling basins and drainage swamp as a breeding site; (2) their ecological roles as grazers (larvae) and semi-aquatic, mid-trophic level predators (juveniles and adults) may allow us to generate larger-scale (ecologically oriented) hypotheses based upon information gathered on them; (3) the grazing habits of larvae may make them particularly susceptible to uptake of sediment-borne pollutants, via ingestion of sediments while feeding; (4) their long larval period (1 to 2 years locally) makes them particularly useful for studies of chronic exposure to pollutants prior to metamorphosis; and (5) they are geographically widespread both naturally and as an introduced species.

Here we summarize recent work conducted on embryonic and larval bullfrogs in the D-Area CCR disposal system. General overviews of the protocols of each study are presented; however, we refer readers to the original publications for detailed descriptions of approaches and analyses. After presenting the approaches and results of prior studies, we synthesize the findings of each study to examine the potential interactions of observed phenomena and the ways that they may affect individuals and populations.

## MORPHOLOGY

Two morphological features of larval bullfrogs have been studied with respect to CCR exposure: the oral disc region and the tail region. Abnormal development of these regions following exposure to contaminants would be predicted to affect the ecology of the individual, either through effects on feeding or swimming, respectively. The initial studies linking morphology of bullfrogs with CCR focused on the oral region, specifically on the presence or absence of complete labial tooth rows. A field survey of populations inhabiting the CCR-polluted site and nearby, unpolluted ponds revealed that abnormalities (defined as at least one tooth row being incomplete) were present in 96 and 85% of tadpoles collected from the settling basin and drainage swamp, respectively, whereas only 3% of tadpoles collected from the reference areas showed signs of abnormalities[12]. Abnormalities were also assessed by comparing the number of labial teeth in two tooth rows from 50 individuals collected from the settling basins and swamp (1 site) and one of the reference sites. In an anterior tooth row, animals from the polluted system had an average of only 5 teeth compared with the reference area in which an average of 50 teeth was present. A similar result was obtained for the posterior row (64 vs. 104 teeth for the pollution-exposed and reference populations, respectively).

These observations[12] provided correlational evidence that oral abnormalities in bullfrogs were related to conditions in the polluted site. However, the design of the initial study did not allow for a causal relationship to be established. A follow-up study was therefore conducted to determine whether the observed abnormalities could be induced by exposure to conditions in the CCR-polluted site[19]. A reciprocal transplant of bullfrog embryos between the polluted and an unpolluted site was conducted so that the effects of environment and maternal/genetic history could be separated with respect to induction of abnormalities. Freshly deposited embryos were collected from the polluted site and an unpolluted site, and groups of embryos from each egg mass were placed into replicate cages in both sites. Tadpoles were collected from all cages and assessed for the presence or absence of oral abnormalities 80 days after hatching. Regardless of the population from which the embryos were derived, 97 to 100% of tadpoles raised in the CCR-polluted site showed abnormalities, whereas less than 1% of those raised in the reference sites were abnormal. Thus, this experiment confirmed that oral abnormalities in larval bullfrogs were induced by environmental conditions in the CCR-polluted site.

While oral abnormalities might be an interesting and easily quantified indicator of environmental problems, a more ecologically important issue is whether or not the abnormalities have important effects on the autecology of affected individuals. We hypothesized that the labial teeth were important for grazing periphyton from solid substrates, and therefore that the absence of labial teeth in abnormal individuals would impair feeding ability and growth when attached periphyton was the primary resource available[12]. The relationship between abnormalities and grazing and growth was addressed in the laboratory, using tadpoles captured from the polluted site and classified as either normal or abnormal. As predicted, the ability to graze periphyton was reduced in abnormal individuals. Tadpoles having oral abnormalities removed less than 1% (per gram wet mass of tadpole) of the periphyton available on plastic substrates, whereas normal tadpoles removed 25%. In a separate study, abnormal and normal individuals (all from the CCR-polluted site) were held for 24 days and fed attached periphyton only, or periphyton plus a particulate source of food. Growth rates were high for both types of animals in the particulate food treatment, indicating that such a resource does not require labial teeth for ingestion. However, in the periphyton-only food treatment, abnormal tadpoles had significantly decreased growth rates compared to normal tadpoles, the former being unable to sustain their initial mass. The laboratory studies suggest that CCR-related abnormalities in the oral region of larval bullfrogs can affect autecology by affecting grazing ability and therefore reducing growth rates[12]. Yet, natural habitats certainly provide many more potential resource types than were present in the laboratory studies, and therefore a strict dependence on periphyton as a sole resource seems unlikely. Thus, caution must be used in extrapolating the results of such laboratory experiments to more natural situations.

In addition to oral abnormalities, axial malformations also occur in larval bullfrogs exposed to CCR. Axial malformations are among the most common, and certainly most conspicuous, of malformations in xenobiotic-exposed amphibian larvae[20,21,22]. In a recent field survey, over 2100 larval bullfrogs (stage 25-35; ref. [23]) were collected from the largest ("primary") settling basin, from the drainage swamp, and from two local reference sites having no history of CCR contamination. In the settling basin and drainage swamp, 36.8 and 17.7% of larvae, respectively, exhibited lateral flexures of the tail[24]. In the two reference sites, 0 and 4.4% of larvae had tail flexures, in agreement with previous reports of the frequency of tail flexures in anurans unexposed to pollutants[20,25].

## BODY BURDENS

Whole body trace element concentrations including As, Cd, Cr, and Se were significantly higher in CCR-exposed populations of bullfrogs exhibiting lateral tail flexures[24]. Some of these trace elements are known teratogens[26,27,28,29] and could be responsible for the observed axial malformations. Concentrations of trace elements such as As, Cd, Cr, and Cu were significantly higher in larvae from the settling basin than in larvae from the polluted swamp[24]. The higher body burdens of trace elements in tadpoles from the settling basin compared to the swamp may explain the observed differences in frequency of axial malformations between the two polluted sites (36.8 and 17.7%, respectively).

## BEHAVIOR

Behavior provides a link between an animal's internal physiology and external environment[30]. Studies in the CCR-contaminated site have focused on behaviors that may affect predation risk. Specifically, initiation of swimming following prodding and swimming speed have been examined as they relate to history of exposure to CCR. Response to prodding and swimming speed over a distance of 1 m were compared for tadpoles captured from the CCR basins and drainage swamp or from a reference pond[31]. Animals of similar sizes from each site were individually placed in a narrow trough in the laboratory and prodded from behind to initiate swimming. Three swimming trials per animal were videotaped for subsequent determination of swimming speed and number of prods required to induce a sustained swim over a 1-m distance. For tadpoles collected from the polluted site, the average swimming speed over 1 m was less than half that of the tadpoles from the reference area. Furthermore, tadpoles from the polluted site were lethargic in their response to a physical stimulus, requiring twice the number of prods to initiate a 1-m swim as did tadpoles from the reference pond. The authors speculated that interactions between accumulated trace elements, structural proteins in the tail, and neurophysiology might explain the results, although these hypotheses were not tested[31].

Response to stimuli and rapid escape are activities that may affect the interaction between a tadpole and a predator. Thus a transplant experiment was conducted to identify the relationship between predation risk and a history of exposure to CCR[31]. Embryos were collected from egg masses laid in an historically unpolluted pond and transplanted into cages in the CCR-polluted site or a reference pond. Sixty-day-old tadpoles from the cages in each site were placed in outdoor tanks that contained or did not contain a predatory, juvenile snapping turtle. After 3 days in the tanks, survival of tadpoles from each site was compared. In the absence of the predator, tadpoles from each site survived well (>97%). However, in the predator treatment, significantly fewer of the CCR-exposed tadpoles survived, compared to reference tadpoles (6 vs. 31 %). Thus, predation risk appeared to be considerably greater for CCR-exposed animals than for reference animals, possibly due to the behavioral differences previously discussed.

The relationship between axial malformations and swimming performance was also examined[24]. Swimming performance of larval bullfrogs without lateral tail flexures from a reference site, the primary settling basin, and the drainage swamp were compared with performance of larval bullfrogs from the settling basin that had lateral flexures. Larvae from the polluted sites (with and without malformations) were up to three times less responsive to physical stimuli than larvae from the reference site[24], as had been reported in the previous study[31]. Moreover, malformed larval bullfrogs from the polluted site were up to 26 and 37% slower than larvae without malformations from the polluted sites and reference site, respectively. Although the ecological significance of hindered swimming performance in malformed larvae remains to be empirically tested, such drastic changes likely affect predation risk (e.g., [31]).

## PHYSIOLOGY

It has been suggested that stressful conditions, such as those experienced in a polluted habitat, might result in an elevation in an individual’s expenditure of energy for maintenance, which represents the basic physiological and activity requirements for survival[32,33,34,35]. Elevated metabolic expenditures may represent costs associated with detoxification mechanisms or other processes invoked to combat effects of contaminants[33]. By examining maintenance energy requirements in individuals, it is possible to infer how energy allocation processes are affected by pollution, and how altered energy allocation in pollution-exposed individuals might affect population-level processes. Energy assimilated from the environment is allocated among the competing processes of maintenance, growth, energy storage, and reproduction. Since a finite amount of energy can be harvested from the environment, an elevation in maintenance expenditures is predicted to detract from the remaining energy available for other processes. Thus, direct effects of pollution on maintenance, via costs incurred at the cellular or biochemical levels (metabolism, biotransformation, and/or sequestration processes), should indirectly affect allocation processes important to population dynamics.

Maintenance energy expenditures, estimated as standard metabolic rate (SMR), were compared for larval bullfrogs based upon their histories of exposure to CCR[13]. Field-collected tadpoles from the polluted site had SMRs 40 to 97% higher than tadpoles collected from a reference site. Measurements made at three temperatures, and during the spring and autumn, consistently showed higher SMRs in individuals inhabiting the CCR-contaminated site.

To overcome the potentially confounding factors associated with comparing physiological parameters in distinct populations, a reciprocal transplant of embryos between the CCR-polluted site and a reference pond was also conducted[13]. The design of this experiment was similar to that employed to determine whether oral abnormalities could be induced due to exposure to CCR during the embryonic and early larval stages[19]. Regardless of the population from which the embryos were derived, maintenance costs at 25 and 80 days posthatching for CCR-exposed individuals were elevated by 39 to 176% over reference animals (depending upon tadpole age and the temperature to which they were acclimatized and analyzed). Interestingly, elevation in SMR in the CCR-polluted system is not restricted only to bullfrogs; increases in SMR have also been found in shrimp (*Palaemonetes paludosus*) transplanted into the CCR-deposition basins[11] and in crayfish (*Procambarus acutus*; [36]) and water snakes (*Nerodia fasciata*; [15]) inhabiting the basins and drainage swamp.

As mentioned above, elevations in metabolic expenditures experienced by bullfrogs and other animals in the CCR-polluted system should detract from energy available for production processes (growth, storage, or reproduction in adult animals). Recent studies suggest that energy storage may be lower in larval bullfrogs collected from the polluted system when compared to animals collected at a reference site (Rowe and Hopkins, unpublished). Average nonpolar (storage) lipid content of tadpoles in the polluted site was about half that of tadpoles in a reference area. However, because lipid content was compared only in field-collected animals, we cannot rule out the possibility that site-specific differences in resource abundance or quality were in part responsible for differences in lipid stores (e.g., [37]). Relationships between CCR-induced elevations in metabolic requirements and other production processes in larval bullfrogs remain speculative at this time, and require empirical investigation.

## SYSTEM-WIDE EFFECTS: HYPOTHESES ABOUT THE INTERACTIONS AMONG OBSERVED PHENOMENA

The studies summarized in this article illustrate that multiple systems are negatively affected in larval bullfrogs exposed to CCR. In all cases, the systems chosen for study were those that have been shown to, or are predicted to, directly or indirectly affect processes at higher levels of organization (see Fig. 1). Morphological abnormalities appear to affect grazing ability and growth rates under conditions of limited resource types, as well as swimming performance. Coupled with reduced ability to respond to stimuli, reduced swimming speed may elevate the risk of predation. Elevation of maintenance energy expenditures, which appears to be common in several species of animals chronically exposed to CCR, must detract from the energy available for processes such as growth and energy storage.

Identification of sublethal responses of bullfrogs provides useful information about some specific traits of individuals that can be modified by exposure to CCR. Yet a more comprehensive view of the overall impacts of inhabiting the polluted environment can be gained by examining the body of information as a whole, such that the potential interplay among affected processes can be examined.

In the following discussion we provide hypotheses about the potential interactions among the observed sublethal effects of CCR exposure that may have deleterious effects on individuals. This discussion is necessarily speculative; it is meant as an exercise to illustrate the complexity in possible responses that can result from modification of multiple systems by pollutants. We recognize that some of the hypothesized interactions would be difficult, if not impossible, to

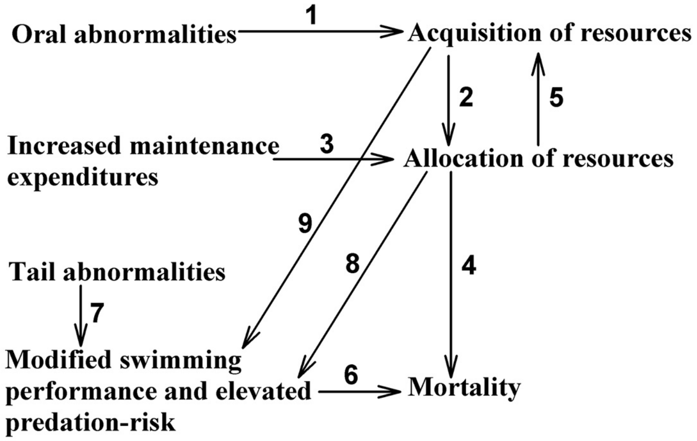

**FIGURE 1.** A diagram summarizing interactions among measured responses and their potential ramifications for larval bullfrogs inhabiting a coal ash–polluted site. Numbered arrows refer to the following measured or hypothesized relationships: 1 = limited ability of individuals with oral malformations to consume periphyton; 2 = decreased energy for allocation to various processes due to limited intake of resources; 3 = modified energy allocation patterns due to high expenditures of energy on maintenance; 4 = mortality resulting from failure to meet maintenance costs; 5 = limited ability to find and procure resources due to limited energy available for foraging activity; 6 = mortality resulting from decreased predator recognition and escape ability; 7 = decreased swimming speed in tadpoles with tail abnormalities; 8 = decreased swimming performance due to limited energy available; 9 = modified foraging behavior in individuals having oral abnormalities resulting from increased search time or area to locate alternative consumable resources.

examine empirically. Our intention is to emphasize the importance of measuring multiple, sublethal responses in pollution-exposed individuals, and to stimulate interest in examining relationships among endpoints that are frequently examined in isolation.

Two of the phenomena observed in the CCR-exposed population of bullfrogs—reduced feeding ability due to oral abnormalities and elevated standard metabolic rates—can have direct ramifications for the energy budgets of individuals. Oral abnormalities appear to reduce the feeding niche of the tadpoles, in effect isolating them from a potentially rich source of energy in the form of periphyton (arrow 1 in Fig. 1). If abundant alternative food sources of required quality are not available, the total per capita resource acquisition may be decreased for tadpoles in the polluted habitat. Decreased acquisition of resources in abnormal animals would therefore reduce the amount of energy that can be allocated to various energy requiring processes (arrow 2 in Fig. 1). Yet, we have also seen that allocation of energy to the maintenance pathway is elevated in CCR-exposed individuals. When considered simultaneously, oral abnormalities and elevated SMR could present a situation in which energy input to the budget is reduced, while energy expenditures are increased (arrow 3 in Fig. 1). If metabolic costs cannot be supported due to severe reductions in acquisition (such that the former exceeds the latter), eventual death of the individual would be predicted (arrow 4 in Fig. 1). On the other hand, if metabolic expenditures are exceeded slightly by total energy acquired and assimilated, the organism may survive, but would have little additional energy to allocate to pathways that compete with maintenance for energy (growth, storage), and to support the energy-demanding process of metamorphosis.

The physiological and morphological impacts observed in our studies may also be important to individuals by affecting foraging energetics. It is possible that the decreased feeding niche experienced by tadpoles with oral deformities results in greater activity and search time in order to find sufficient consumable resources. Feeding efficiency would therefore be reduced, since searching for adequate food would require some additional expenditure of energy. Yet elevated metabolic expenditures in individuals from the polluted site might reduce the net energy available to support foraging activities, such as searching and food handling. Thus, tadpoles inhabiting the polluted site could lack the stores of energy required to locate foraging sites and acquire necessary resources, exacerbating the energetic dilemma (arrow 5 in Fig. 1).

The swimming performance and predator avoidance study[31] showed that decreased swimming speed and recognition of a stimulus correlated with low survival of tadpoles from the polluted site when a predator was present (arrow 6 in Fig. 1). Thus mortality of tadpoles due to predation could be higher than normal in the polluted habitat due to modified behavior of tadpoles. We also expect an elevated predation risk in tadpoles with abnormal tails, which likewise had decreased swimming speeds (arrow 7 in Fig. 1; [24]). It was speculated that modified behaviors might be related to neurotoxic properties of the contaminant mixtures, or possibly incorporation of trace elements into structural proteins[31]. However, it is also possible that decreased energy acquisition or increased metabolic expenditures by tadpoles in the polluted site results in little energy available for activities necessary for avoiding predators, increasing predatory impacts on the population (arrows 8 and 9 in Fig. 1). Predation risk could also be influenced by modified energy acquisition or allocation (arrows 8 and 9 in Fig. 1) by requiring that a greater than normal amount of time be spent foraging or moving among foraging sites.

## MORTALITY

Most work on bullfrogs and CCR pollution has been directed at identifying sublethal impacts on individuals, yet it is important to note that survival of bullfrogs through the early larval period (80 days posthatching) was lower in the polluted site (15%) than in a reference site (67%; [13]). Furthermore, those few larvae that survive the early larval period do so with the variety of physiological, morphological, and behavioral impairments described above. The cumulative effects of exposure to CCR and predators throughout the extended larval period of the bullfrog (>1 year) probably further reduces the number of tadpoles that can eventually complete metamorphosis and be recruited from the polluted aquatic environment. We have occasionally observed recently metamorphosed individuals in the polluted area, indicating that some individuals are capable of completing the premetamorphic period (Rowe and Hopkins, unpublished data). However, given the multiple stresses incurred by individuals that survive the early larval period, recruitment of juveniles from the coal-ash polluted habitat must be reduced compared to populations in unpolluted areas (e.g., [37]).

## SUMMARY

Several individual-level responses have been reported for larval bullfrogs inhabiting a site contaminated by trace element–rich CCR inputs. Responses include morphological changes that influence resource harvesting, growth, and swimming activities, behavioral changes that appear to increase susceptibility to predators, and physiological changes that may reduce an individual’s ability to allocate energy to production processes. Any of these responses on their own may have overall significance for health or fitness of affected individuals, yet when the responses are integrated, an even more severe situation emerges. An integrated perspective on contaminant effects on individuals requires that endpoints such as behavior, physiology, and morphology/development be widely incorporated into studies of environmental contamination. Such endpoints themselves integrate multiple lower-order processes (molecular, cellular, and organ-level changes) and thus represent cumulative effects of many disparate actions of the contaminants. Furthermore, because environmental contaminants frequently occur in complex mixtures of compounds having unique individual modes of action along with synergistic and antagonistic interactions, we must expect that numerous features of individuals will be affected in contaminated habitats. Additionally, the interdependency of biological processes operating to form a functioning individual suggests that rarely, if ever, will one system be affected by contaminants independently of others.

## ACKNOWLEDGMENTS

This manuscript benefited from the suggestions of W. Dunson, D. Sparling, M. Hall , and S. Kelly. Support for the preparation of this article was provided by grant R827581 from the U.S. Environmental Protection Agency's STAR-Futures Program and by U.S. Department of Energy Financial Assistance Award Number DE-FC09-96SR18546 to the University of Georgia Research Foundation.
